# A nomogram for predicting postoperative pulmonary infection in esophageal cancer patients

**DOI:** 10.1186/s12890-021-01656-7

**Published:** 2021-09-06

**Authors:** Shuang Li, Jingwen Su, Qiyu Sui, Gongchao Wang

**Affiliations:** School of Nursing and Rehabilitation, Shandong University, Jinan, 250012 China

**Keywords:** Esophageal cancer, Postoperative pulmonary infection, Nomogram

## Abstract

**Background:**

Although postoperative pulmonary infection (POI) commonly occurs in patients with esophageal cancer after curative surgery, a patient-specific predictive model is still lacking. The main aim of this study is to construct and validate a nomogram for estimating the risk of POI by investigating how perioperative features contribute to POI.

**Methods:**

This cohort study enrolled 637 patients with esophageal cancer. Perioperative information on participants was collected to develop and validate a nomogram for predicting postoperative pulmonary infection in esophageal cancer. Predictive accuracy, discriminatory capability, and clinical usefulness were evaluated by calibration curves, concordance index (C-index), and decision curve analysis (DCA).

**Results:**

Multivariable logistic regression analysis indicated that length of stay, albumin, intraoperative bleeding, and perioperative blood transfusion were independent predictors of POI. The nomogram for assessing individual risk of POI indicated good predictive accuracy in the primary cohort (C-index, 0.802) and validation cohort (C-index, 0.763). Good consistency between predicted risk and observed actual risk was presented as the calibration curve. The nomogram for estimating POI of esophageal cancer had superior net benefit with a wide range of threshold probabilities (4–81%).

**Conclusions:**

The present study provided a nomogram developed with perioperative features to assess the individual probability of infection may conducive to strengthen awareness of infection control and provide appropriate resources to manage patients at high risk following esophagectomy.

## Introduction

Esophageal cancer (EC) was diagnosed with 572,034 new cases and results in 508,585 deaths around the world in 2018 [1]. The past few decades have seen a rapid increase in the incidence of esophageal cancer [2]. Although multimodal therapy is composed of surgical treatment, radiotherapy, and chemotherapy, prognosis outcomes remain poor for EC, with only 15–25% of patients surviving beyond 5 years [3]. For patients with esophageal cancer, esophagectomy remains the primary option for esophageal cancer patients because of removing the tumors of the esophagus and improving symptoms. Despite the incidence and mortality of esophageal cancer decline resulting from medical advances and perioperative management, postoperative pulmonary complications, especially postoperative pulmonary infection (POI), which is a principal problem associated with patient’s prognosis and outcomes [4]. Several studies reported that POI occurred in almost 16–40% of EC patients and has been identified to be a factor for perioperative death and long-term survival [5–7]. It needs to, therefore, distinct and identify those patients at the greatest risk of POI, and promote early intervention to reduce its incidence or improve postoperative prognosis outcomes. A study observed that the increasing POI rate was connected with several risk factors such as age, smoking, preoperative comorbidity, lower hemoglobin, higher creatinine, postoperative dysphagia [8–10]. However, the risk factors of POI following esophageal cancer resection are inconclusive, showing the differences in institutions and healthcare delivery facilities.

Construct a mathematical model to predict POI may be a solution to the issue. A nomogram is a graphical depiction that presents a regression model in a friendly manner and simplifies risk assessment, offering healthcare practitioners a user-friendly interface to map the probability of an event to individual patients and enhancing clinical decision-making of both medical personnel and patients [11, 12]. Such a device would strengthen the validity and objectivity of risk assessment. Therefore, this study seeks to develop and validate a nomogram for predicting POI with perioperative information.

## Methods

### Study population

This retrospective cohort study involved adult patients with a newly diagnosed EC from January 1, 2018, to December 31, 2019 at the Shandong Provincial Hospital. Study samples and treatment data were retrieved from the database of respective surgical departments. Patients will be included in this study if they meet the following requirements: (1) aged 18 years or older (2) pathological section diagnosed as malignant esophageal cancer (3) underwent curative esophagectomy. On the other hand, if EC patients who died within 24  hours after surgery or lack complete case records were excluded. The data used in this study was approved by the Institutional Review Board of Shandong University, and was exempt from the requirement for individual patient consent because contained no personal identifiers. The study complied with the principles of Declaration of Helsinki.

### Study outcomes and data collection

The primary endpoint POI, definition referred to Centers for Disease Control and Prevention and National Healthcare Safety Network surveillance definition [13]. Pathological staging was performed according to the American Joint Committee on Cancer (AJCC) Staging Handbook (7th edition) [14].The following data were collected: gender, age, length of stay, body mass index, smoking, drinking, hypertension, diabetes mellitus, coronary heart disease, chronic obstructive pulmonary disease, pulmonary tuberculosis, tumor type, primary tumor site, AJCC pathological stage, AJCC clinical stage, chemoradiotherapy, lymph node metastasis, forced vital capacity, forced vital capacity percentage predicted, forced expiratory volume in one second, forced expiratory volume in one second percentage predicted,  albumin, hemoglobin, pattern of anastomosis, surgery time, intraoperative bleeding, perioperative blood transfusion, American Society of Anesthesiologists score, postoperative pulmonary infection.

### Statistical analysis

Continuous variables were reported as mean with standard deviation (SD) and categorical variables as frequency with percentage. The Least absolute shrinkage and selection operator (LASSO) regression model was performed to tackle the collinearity of candidate variables to select the optimal predictive variables [15]. Multivariable logistic regression analysis was generated using selected predictors from LASSO analysis. The features were presented as odds ratio (OR) and 95% confidence interval (CI). A two-tailed P value < 0.05 was considered statistical significance. Model discrimination was assessed by concordance index (C-index) and calibration was evaluated by calibration curve. Decision curve analysis (DCA) was adopted to determine the clinical usefulness and net benefit of the nomogram [16]. Externally validation was generated to confirm the stability of the nomogram in the validation cohort using 1000 bootstrap resamples and calculating a relatively corrected C-index. Statistical analyses were carried out using SPSS, version 25.0 and R Studio, version 4.0.2.

## Results

### Population characteristics

Table 1 shows the clinical characteristics of the study population. A total of 637 EC patients were enrolled in this study, separated by training cohort (from January 1, 2018, to July 31, 2019) and validation cohort (from August 1, 2019, to December 31, 2019). Of 446 patients (mean [SD] age, 59.77 [8.3] years; 349 men [78.3%]) in training cohort, 95 patients (21.3%) were diagnosed POI, while 191 patients (mean [SD] age, 58.47[8.7] years; 170 men [89.0%]) composed of validation cohort, 36 patients (18.8%) experienced POI.

**Table 1:** Characteristics of the study population

Variable	Training cohort n = 446 (%)	Validation cohort n = 191 (%)
Gender		
Male	349 (78.3)	170 (89.0)
Female	97 (21.7)	21 (11.0)
Age, X(SD), years	59.77 (8.3)	58.47 (8.7)
Length of stay, X(SD), days	23.89 (15.2)	24.07 (12.0)
Body Mass Index, X(SD), (kg/m^2^)	23.19 (3.0)	22.87 (2.8)
Smoking		
Yes	291 (65.2)	131 (68.6)
No	155 (34.8)	60 (31.4)
Drinking		
Yes	283 (63.5)	124 (64.9)
No	163 (36.5)	67 (35.1)
Hypertension		
Yes	91 (20.4)	52 (27.2)
No	355 (79.6)	139 (72.8)
Diabetes mellitus		
Yes	38 (8.5)	16 (8.4)
No	408 (91.5)	175 (91.6)
Coronary heart disease		
Yes	22 (4.9)	11 (5.8)
No	424 (95.1)	180 (94.2)
COPD		
Yes	3 (0.7)	0 (0)
No	443 (99.3)	191 (100)
Pulmonary tuberculosis		
Yes	27 (6.1)	13 (93.2)
No	419 (93.9)	178 (6.8)
Tumor type		
Squamous cell carcinoma	419 (93.9)	181 (94.8)
Adenocarcinoma	14 (3.1)	6 (3.1)
Other	13 (3.0)	4 (2.1)
Primary tumor site		
Upper	26 (5.8)	9 (4.7)
Middle	242 (54.3)	108 (56.6)
Lower	137 (30.7)	55 (28.8)
Others	41 (9.2)	19 (9.9)
AJCC pathological stage		
1	24 (5.4)	11 (5.8)
2	339 (76)	136 (71.2)
3	82 (18.4)	44 (23)
4	1 (0.2)	0 (0)
AJCC clinical stage		
1	50 (11.2)	22 (11.5)
2	225 (50.4)	99 (51.9)
3	171 (38.4)	69 (36.1)
4	0 (0)	1 (0.5)
Chemoradiotherapy		
Yes	1 (0.2)	0 (0)
No	445 (99.8)	191 (100)
Lymph node metastasis		
Yes	197 (44.2)	87 (45.5)
No	249 (55.8)	104 (54.5)
FVC, X(SD), L	3.45 (7.4)	5.18 (27.1)
FVC% pred, X(SD), %	89.36 (14.7)	90.93 (15.4)
FEV1, X(SD), L	2.70 (0.7)	2.77 (0.7)
FEV1% pred, X(SD), %	98.16 (27.2)	97.39 (18.4)
Albumin (g/L), X(SD)	33.34 (5.1)	32.40 (3.8)
Hemoglobin (g/L), X(SD)	124.15 (62.1)	120.69 (17.8)
Pattern of anastomosis		
1	193 (43.3)	82 (42.9)
2	121 (27.1)	49 (25.7)
3	44 (9.9)	13 (6.8)
4	88 (19.7)	47 (24.6)
Surgery time, h		
≤ 3	153 (34.3)	62 (32.5)
> 3	293 (65.7)	129 (67.5)
Intraoperative bleeding, X(SD), ml	183.36 (120.3)	213.98 (159.8)
Perioperative blood transfusion		
Yes	271 (60.8)	60 (31.4)
No	175 (39.2)	131 (68.6)
ASA score		
1	63 (14.1)	27 (14.1)
2	349 (78.3)	155 (81.2)
3	34 (7.6)	9 (4.7)
Postoperative pulmonary infection		
Yes	95 (21.3%)	36 (18.8%)
No	351 (78.7%)	155 (81.2%)

*COPD* chronic obstructive pulmonary disease, *AJCC* American Joint Committee on Cancer, *FVC* forced vital capacity, *FVC% pred* forced vital capacity percentage predicted, *FEV1* forced expiratory volume in one second, *FEV1% pred* forced expiratory volume in one second percentage predicted, *ASA* American Society of Anesthesiologists

### Selected predictors

**Fig. 1:**
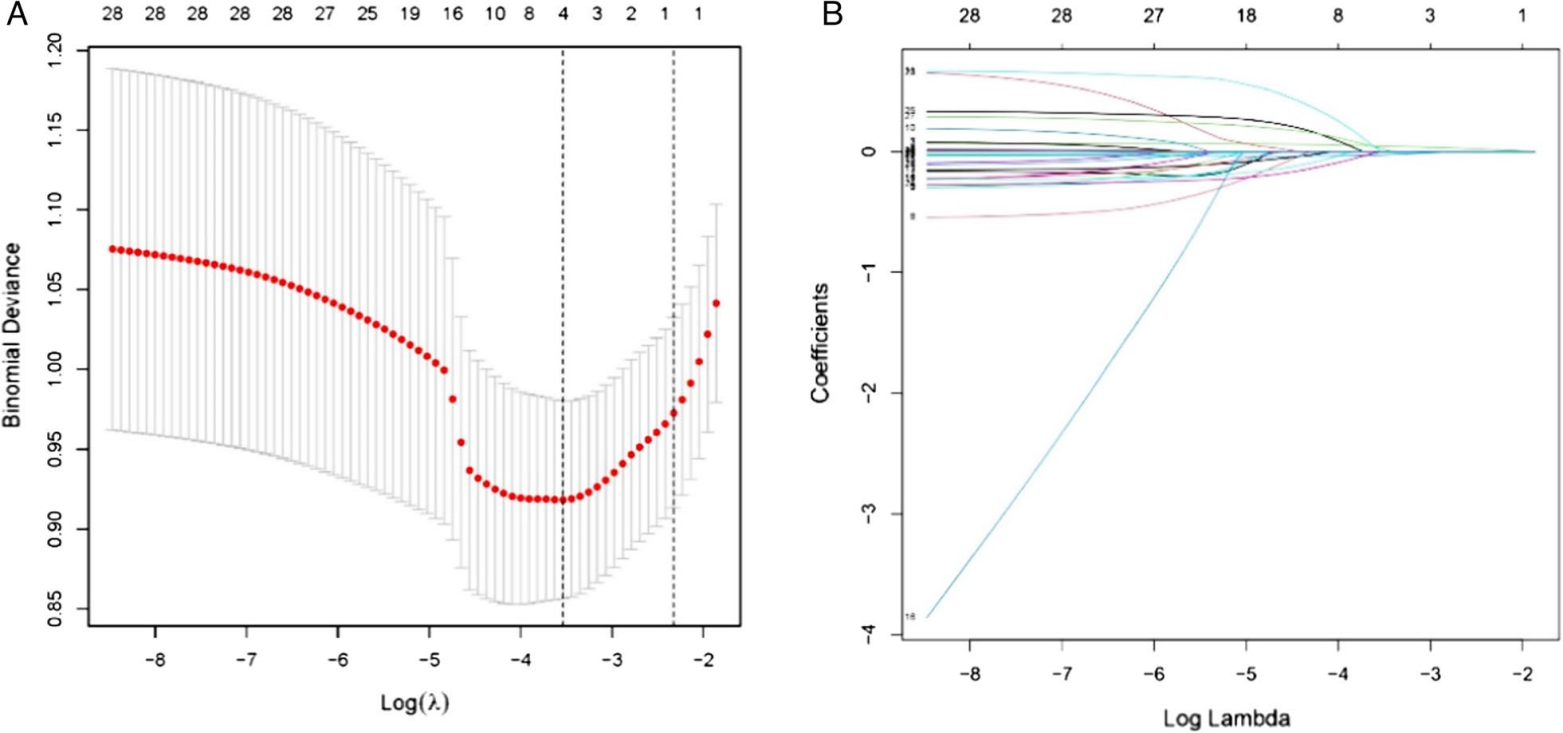
Perioperative variable selection using a LASSO logistic regression model. (**a**) Dotted vertical lines were depicted at the optimal values by using the minimum criteria (lambda.min) and 1 SE of the minimum criteria (lambda.1se). (**b**) LASSO coefficient profile of 28 variables. The coefficient profile is plotted according to the logarithmic sequence. Five-fold cross-validation via minimum criteria was used to determine the optimal predictors of model resulted in four features with nonzero coefficients

Of 28 features, 4 potential predictors were finally selected on the basis of LASSO regression analysis (Fig. 1). The optimal predictors incorporated length of stay, albumin, intraoperative bleeding, perioperative blood transfusion. Multivariable logistic regression analysis based on four predictors screened from LASSO regression analysis was carried out to create the final model (Table 2).

**Table 2:** Prediction factors for the risk of postoperative pulmonary infection with esophageal resection

Intercept and variable	β	Odds ratio (95% CI)	p value
Intercept	− 1.91	0.15 (0.012–2.017)	0.148
Length of stay	0.07	1.07 (1.046–1.101)	< 0.001
Albumin	− 0.06	0.94 (0.879–1.004)	0.085
Intraoperative bleeding	0.00	1.00 (1.001–1.005)	0.004
Perioperative blood transfusion	0.28	1.32 (0.730–2.438)	0.360

### Construction and validation of the nomogram

**Fig. 2:**
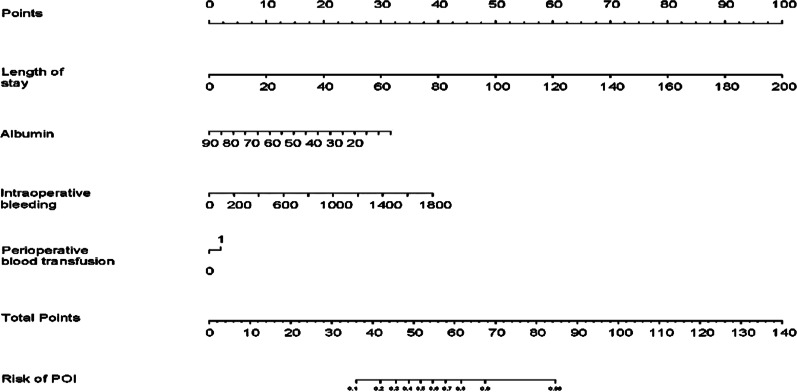
Nomogram for prediction of POI in esophageal cancer patients underwent curative operation

**Fig. 3:**
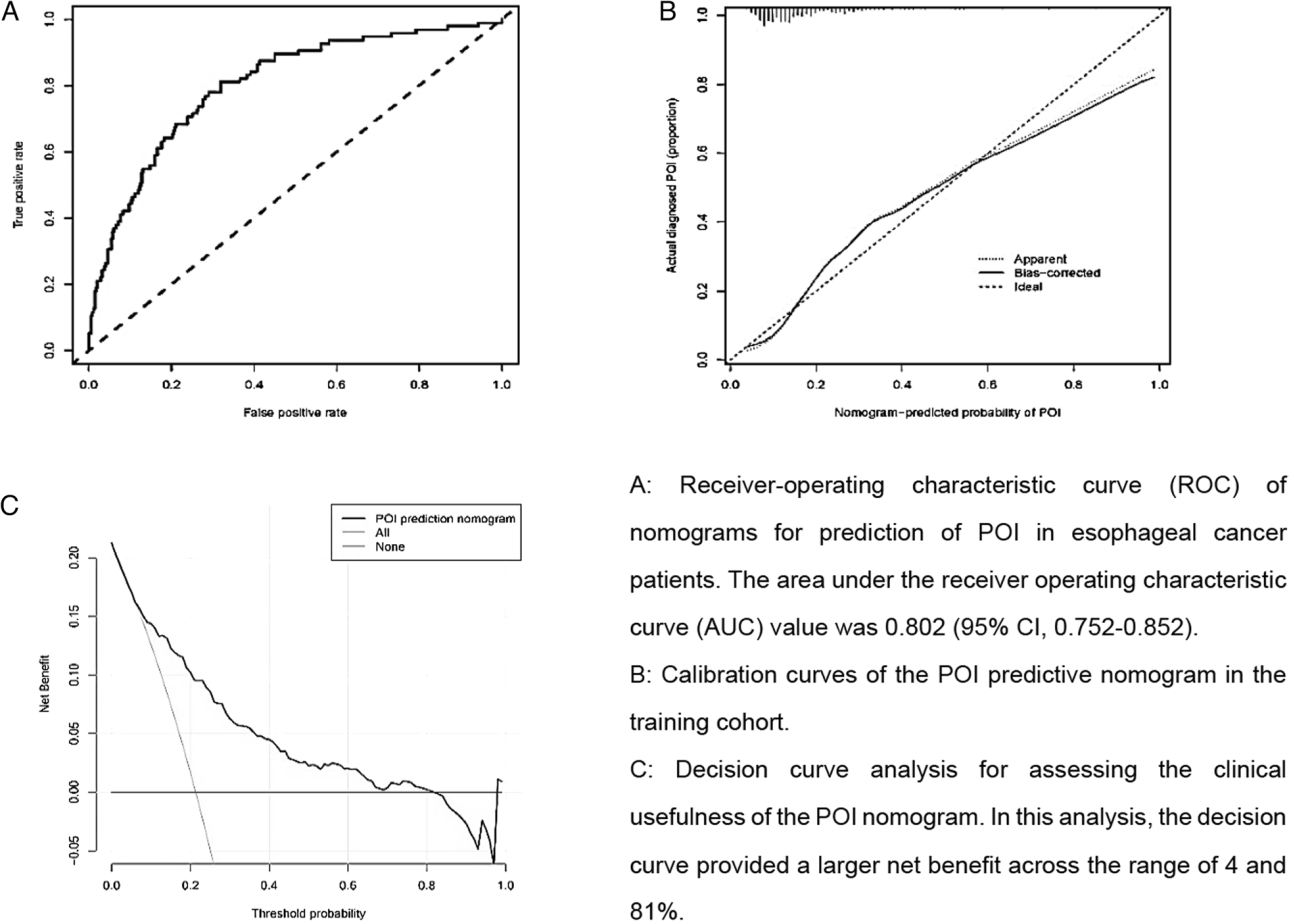
Evaluation of the nomogram for prediction of POI in esophageal cancer patients who underwent esophagectomy

The nomogram for predicting POI in esophageal cancer patients who underwent curative operation was shown in Fig. 2. Model discrimination, as quantified by the C-index, was 0.802 (95% CI 0.752–0.852), indicating the predictive model can better distinguish POI patients from non-POI patients (Fig. 3a). The calibration plot (Fig. 3b) demonstrates good consistency between the predicted risk of POI and the observed actual risk. The clinical value of the nomograms was assessed by decision curve analysis on the basis of the net benefit and threshold probabilities. As for POI of esophageal cancer, the graph (Fig. 3c) suggested the nomogram had superior net benefit with a wide range of threshold probabilities (4–81%). To confirm the stability of the model, we externally validate the nomogram generated in the training cohort. The validation cohort comprised 191 esophagectomy patients from August 1, 2019, to December 31, 2019. The predictive nomogram for assessed individual risk of POI, when applied to the validation cohort with a C-index of 0.763 (95% CI 0.669–0.857).

## Discussion

In this study, the incidence of POI was 20.6%, which is comparable to previous findings [17, 18]. The multivariable regression analysis of this retrospective study showed that length of stay, albumin, intraoperative bleeding, and perioperative blood transfusion were independent factors of POI. Identification of destructive and protective factors as well as perioperative management with care delivery can facilitate the control of POI, which furthers clinical prognosis and overall survival [17, 19].

The current study found that length of stay (LOS) accounts for the increasing incidence of POI. This result is in accordance with earlier observation, which showed that extra length of stay attributable to the possibility of developing infection [20]. Prior researchers examined the relationship between LOS and healthcare-associated infection, they found that 1,039 samples of 51,691 patients experienced respiratory infection. A possible explanation for these results is likely to be related to patients with prolonged LOS may undergo bacterial colonization [21].

Our study found that albumin was clinically relevant to the occurrence of respiratory infection. Consistent with the current result, previous study has demonstrated that the rate of respiratory infection occurred in patients with hypoalbuminemia after radical esophagectomy obviously increased [22]. This result may be explained by the fact that hypoalbuminemia contributes to the decrease of plasma osmotic pressure, which induces pulmonary interstitial edema. In addition, reduced gas dispersion and abnormal ventilation to blood flow ratio make pulmonary infections appeal to occur in patients with esophageal cancer. On the other hand, hypoalbuminemia patients were exposed to pulmonary infection by impairing the immunity of patients, which was frequently observed in patients who underwent resection. Moreover, previous researches have noted that hypoalbuminemia played a great role in the prediction value for the mortality and morbidity rates in esophageal cancer population [23].

The multivariate regression analysis revealed that respiratory infection after esophagectomy could be attributed to intraoperative bleeding. With respect to respiratory infection after curative esophagus surgery, several researchers observed that higher blood loss was identified as an independent risk factor for pulmonary infection [19, 24]. Furthermore, for patients with respiratory infection after esophagectomy, greater blood loss was an important indicator of mortality. Increased intraoperative blood loss has been shown to be associated with an increasing incidence of pulmonary complications and hospital death after esophagectomy [25].

In this study, perioperative blood transfusion was found to responsible for POI. There are similarities between the attitudes expressed by researchers in prior studies, they all agreed on the correlation between blood transfusion and increasing susceptibility to pulmonary infection [26, 27]. Perioperative blood transfusion may work collaboratively with operational stress to induce immunosuppression, which was considered as potential mechanisms of increasing in pulmonary infection following esophagectomy [28].

This retrospective study suggested that a nomogram developed with perioperative data to generate personalized evaluates of postoperative pulmonary infection following esophagectomy may distinguish target patients at high risk of pulmonary infection. For example, if a patient was hospitalized for 60 days, had transfused blood, had an albumin level of 25, and had intraoperative bleeding of 800 ml, his total score is approximately 72.5 points corresponded to approximately 93% risk of POI.

This study is subject to certain limitations. In this retrospective study, the type of specific-infected bacteria could not be certain. Moreover, the additional disadvantage of this study was the limited sample of participants. Additionally, the study is limited by the lack of information on sufficient variables. Some potential variables are not accessible in the database, such as patient-related factors (economic status, social support, education level, health knowledge) and perioperative factors (anesthesia method, Intubation method, medication status, other complications).

## Conclusions

This research identified that length of stay, albumin, intraoperative bleeding, and perioperative blood transfusion emerged as reliable predictors of POI. The findings indicated that the patient-specific nomogram with external validation may have important implications for paying much attention to EC patients with postoperative infection and help decrease the occurrence of postoperative infection cases. Further studies, which take other clinically-relevant variables into account, will perfect the nomogram.

## Data Availability

The datasets are available from the corresponding author on reasonable request.

## Abbreviations

EC: Esophageal cancer
POI: Postoperative pulmonary infection
LASSO: Least absolute shrinkage and selection operator
OR: Odds ratio
CI: Confidence interval
C-index: Concordance index
DCA: Decision curve analysis
ROC: Receiver operating characteristics
AUC: Area under the curve
